# The long-run effects of government expenditure on private investments: a panel CS-ARDL approach

**DOI:** 10.1007/s12197-023-09617-y

**Published:** 2023-02-16

**Authors:** Gianni Carvelli

**Affiliations:** Department of Economic and Social Sciences, Catholic University of the Sacred Heart, Piacenza, Italy

**Keywords:** CS-ARDL, Fiscal policy, Government expenditure, Panel data, Private investments, C33, E22, E62, H50, H60

## Abstract

We re-explore the link between government expenditure and private investments within a modern econometric framework. Performing a dynamic analysis on a panel of 28 OECD countries over 1990–2019, we account for nonstationarity, country-heterogeneity, and cross-sectional dependence. By estimating an ECM version of the novel CS-ARDL model, we find robust evidence of both short-run and long-run adverse effects of aggregate government expenditure on private investments. Increases in *productive* expenditure have no significant effect on the long-run dynamics of private investments, whereas reallocating public resources towards *productive* expenditure enhances them. By contrast, both level increases and shifts of resources towards *unproductive* expenditure discourage capital formation in the private sector. Using the government budget constraint (GBC) system, we observe that the effects of government expenditure depend on how it is financed. In most cases, the short-run fall in private investments is mainly associated with tax-financed expenditure, while debt-financed expenditure appears to be the most detrimental in the long-run.

## Introduction

The current global economic disruption induced by the Covid-19 pandemic and the war in Ukraine has revived the debate on the effects and sustainability of fiscal stimuli. Governments are massively resorting to expansionary policies, and such interventions will likely persist.^1^ However, the long-term effects of increased public debt and the allocation of public resources represent concerns that require a careful assessment. Such issues appear to be even more relevant today as central banks are raising interest rates to cope with the drastic surge in the inflation rate. The existing literature offers several insightful studies that involve the analysis of the GDP effects of aggregate government expenditure. Some focus more specifically on the effects of single or grouped components of government expenditure. For the latter, the common practice is to build the analyses on the seminal papers of Barro (1990) and Devarajan et al. (1996), who categorise as *productive* those spending components that are likely to stimulate long-run aggregate growth thanks to their effects on the private sector and as *unproductive* the remaining part. In addition, some studies encompass the way government expenditure is financed (e.g., Kneller et al. 1999; Gemmell et al. 2011; Gemmel et al. 2016). Although government spending is typically associated negatively with economic growth, the results appear to be intensely dependent on the level of development of the economies, the type of public goods or services financed, and the method of financing. However, most of the existing studies focus on GDP effects. Less attention was dedicated to the direct effects of government expenditure on the private sector. Even less effort was devoted to analysing the effects of productive and unproductive government expenditure on private investments. Although the existing studies provide interesting insights (see, for instance, Giannaros et al. 1999; Ahmed and Miller 2000; Furceri and Sousa 2011; Afonso and Aubyn 2019), normally linking negatively public spending to private investments, we believe that further contribution in the field is worth. In fact, considering that the current historical period is characterised by substantial increases in public spending and deficits, the development of new analyses on recent time series might offer novel research directions and policy recommendations. Another argument that motivates us to focus on private investments is that the dynamics of private capital formation do not necessarily follow the dynamics of aggregate output, especially in periods characterised by massive government interventions. For instance, Blanchard and Perotti (2002) argue that increases in government spending lead simultaneously to a positive output response and a sizeable investment fall.^2^ One could argue that a relevant share of the increase in GDP that follows additional government spending is due to how the former is mathematically constructed, regardless of its effects on economic activity.^3^ Econometrically, this translates into endogeneity of government expenditure when estimating its impact on GDP. In addition, if GDP is employed as the dependent variable and tax revenue is controlled for, the impact of government expenditure on economic growth is overestimated.

One of the main rooms for contributing to the existing applied literature lies in the econometric approach. As Eberhardt (2012) points out, the applied macroeconomics literature is dominated by estimators originally developed for micro panel data – which normally assume that the errors are not cross-sectionally correlated – although the existence of cross-sectional dependence typically characterises macro datasets. Cross-sectional dependence arises when the units are simultaneously affected by global shocks or local spillovers. These common factors are generally unobservable. Neglecting them does not lead to a mere omitted variable problem as they rather represent a set of latent drivers of the economy (Eberhardt and Teal 2013; Eberhardt and Presbitero 2015). Assuming cross-sectional independence when it does not hold might lead to several sources of biases (Phillips and Sul 2003; Andrews 2005; Everaert and De Groote 2016). The recent econometric literature on macro panel data – often referred to as *second-generation panel data econometrics* – addresses in various ways the issue of cross-sectional dependence^4^ whose methods largely rely on estimators designed to accommodate individual-heterogeneity like the flexible Pesaran and Smith (1995) 's Mean Group (MG) and Pesaran et al. (1999) 's Pooled Mean Group (PMG) estimators. An interesting study in which country-heterogeneity is addressed within a dynamic panel framework is performed by Calderón et al. (2015), who estimate an infrastructure-augmented production function. As it concerns identification strategy and the economic research question, the closest analysis to the present article can be found in a recent work by Carvelli (2022), which focuses on the long-run response of private investments to variations in the levels of the single components of government expenditure. However, as we will see in the subsequent sections, the present study addresses different economic aspects of the phenomenon. The contributions to the related literature can be summarised as follows.

Firstly, we explore short-run dynamics and long-run effects of aggregate government expenditure on private investments within a dynamic panel framework that accommodates nonstationarity, country-heterogeneity, and cross-sectional dependence. As we will see afterwards, the hypothesis tests on cross-sectional dependence and unit root lead us to employ an error correction model (ECM) version of the cross-sectional autoregressive distributed lag model (CS-ARDL) developed by Chudik et al. (2016).

Secondly, we consider a bipartition of government expenditure into *productive* and *unproductive*, classified according to socioeconomic objectives. By applying such a bipartition, we can estimate the impact on private investments of level increases in the two spending categories and the effect of a mere reallocation of public resources towards a given category, therefore focusing on the composition of government expenditure.

Thirdly, we assess whether the relationship between government expenditure and private investments depends on how public spending is financed. By including the government budget constraint (GBC) system proposed by Miller and Russek (1997), we condition the estimates to various fiscal combinations to allow for variations in the methods of financing.

## Data and preliminary tests

We start our analysis by assuming the following deterministic investment function:

1 $$y=f({\varvec{x}},{\varvec{w}})$$

where *y* is the private investment, ***x*** represents the set of fiscal variables of interest, and ${\varvec{w}}$ denotes a set of determinants of private investments. The fiscal variables include government expenditure at disaggregate levels, tax revenue, and primary deficit.^5^ The covariates contained in the vector ${\varvec{w}}$ are *i*) the aggregate output per capita, to control for the relative size of the economies and the ties between economic growth and expected profitability of investments, and *ii*) real interest rate, as it represents the cost of investment and is closely tied to monetary policy. Such determinants of investments are selected following Mody and Murshid (2005), Afonso and Aubyn (2009), Ashraf and Herzer (2014), Afonso and Aubyn (2019), and Chu et al. (2020). As we will see later, our empirical framework requires a parsimonious model specification. Further covariates will be included as a robustness exercise (Section 6).

We exploit the recently updated Government Financial Statistics (GFS) database from the International Monetary Fund (IMF), which provides government expenditure data at aggregate and disaggregate levels and other fiscal data like tax revenue and deficit/surplus. Data on private investment are gathered from the AMECO database of the European Commission's Directorate General for Economic and Financial Affairs. We furthermore collect data on GDP and real interest rate on 10-year government bonds from the Penn World Table database, while data on international trade come from the World Developing Indicator (WDI) of the World Bank. Data are collected at the annual level over 1990–2019 for 28 OECD countries.

We apply a bipartition of total government expenditure into *productive* and *unproductive*. Following Barro (1990), Devarajan et al. (1996), Bleaney et al. (2001), Adam and Bevan (2005), Park (2006) and Christie (2014), and Chu et al. (2020), we consider the following classifications: *productive* government expenditure as the sum of spending on education, health, defence, housing & community amenities, economic affairs, general public services, and environment; *unproductive* government expenditure as the sum of spending on culture, social protection and safety & public order.

Table 1 reports the descriptive statistics that will be employed in the baseline regressions. We consider the partitions of government expenditure as shares of GDP and total government expenditure (TGE). From one side, with the inclusion of the GDP share of government expenditure in the model, we can observe the elasticity of private investments to an increase in expenditure. On the other side, considering the TGE shares of the expenditures allows us to analyse the effect of a mere reallocation of public resources from one category to another (Devarajan et al. 1996; Gemmell et al. 2016; Chu et al. 2020).

**Table 1:** Descriptive statistics

Symbols	Description	N*T	Mean	Min	Max
INV	Logarithm of real private investments per capita	800	8.418	6.013	10.320
TGE / GDP	Total government expenditure as a share of GDP	809	0.433	0.188	0.651
PGE / GDP	Productive government expenditure as a share of GDP	775	0.248	0.138	0.438
UGE / GDP	Unproductive government expenditure as a share of GDP	775	0.184	0.088	0.286
PGE / TGE	Productive government expenditure as a share of total government expenditure	775	0.568	0.486	0.764
UGE / TGE	Unproductive government expenditure as a share of total government expenditure	775	0.416	0.236	0.506
TAX	Tax revenue as a share of GDP	1000	0.401	0.159	0.613
DEFICIT	Cyclically adjusted primary deficit as a share of GDP	896	-0.006	-0.165	0.150
GDP	Logarithm of real GDP per capita	1080	10.359	8.975	11.481
INTEREST	Real interest rate on 10-year government bonds	788	4.922	-0.525	23.917

We investigate the presence of cross-sectional dependence by conducting the CD test proposed by Pesaran (2021). The CD statistic converges in distribution to a standard normal probability density function under the null hypothesis of no cross-sectional dependence. Such a test is appealing because it allows for nonstationarity, slope heterogeneity, and structural breaks. Monte Carlo simulations show that the test likely performs well in small datasets (for both *N* and *T* small). According to the results reported in Table 2, there is strong evidence against the null hypothesis of no cross-sectional dependence for all the variables considered.

**Table 2:** CD test

	ρ	|ρ|	CD stat	*p*-value		ρ	CD stat	*p*-value
INV	0.581	0.627	59.130	0.000	UGE / TGE	0.249	26.380	0.000
TGE / GDP	0.275	0.384	30.260	0.000	TAX	0.051	6.550	0.000
PGE / GDP	0.324	0.412	33.600	0.000	CAPB	0.319	37.490	0.000
UGE / GDP	0.251	0.418	26.740	0.000	GDP	0.908	124.800	0.000
PGE / TGE	0.237	0.402	25.180	0.000	INTEREST	0.800	80.770	0.000

Notes: Under the null hypothesis of cross-section independence, CD ~ *N*(0,1). The parameter ρ measures the average cross-sectional correlations

Since cross-sectional dependence is present in our panel, we shall select the panel unit root test accordingly. Im et al. (2003) propose the so-called IPS test that allows the autoregressive parameters to vary across the units. This procedure is extended by Pesaran (2007), who proposes the cross-sectionally augmented IPS test (or CIPS test). The CIPS test accommodates the existence of cross-sectional dependence by augmenting the ADF regressions with current and lagged values of the variables. Under the null hypothesis of nonstationarity of the time series, the CIPS test statistic has a non-standard distribution. The related results (reported in Table 3) denote the existence of a mixed order of integration, as the variables are either *I*(0) or *I*(1).

**Table 3:** CIPS test

	H0: all series within the panel are nonstationary Ha: some series within the panel are stationary			
	Levels		First differences	
	*Zt*-bar (1)	*p*-value (2)	*Zt*-bar (3)	*p*-value (4)
INV	0.106	0.542	–6.531	0.000
TGE / GDP	-0.418	0.338	–10.939	0.000
PGE / GDP	1.040	0.851	–2.571	0.005
UGE / GDP	1.880	0.970	–3.313	0.000
PGE / TGE	-2.490	0.006	–9.992	0.000
UGE / TGE	-2.465	0.007	–9.558	0.000
TAX	-0.472	0.319	–10.325	0.000
CAPB	-2.317	0.010	–8.464	0.000
GDP	0.926	0.823	–3.536	0.000
INTEREST	-3.704	0.000	–10.181	0.000

Notes: The optimal lag length is selected according to the Akaike Information Criterion (AIC). The intercept and linear time trends are included in the equations. Columns 1 and 3 report the test statistics, whereas the related *p*-values are reported in columns 2 and 4. The first (second) two columns refer to the model in levels (first differences)

## Model specification and econometric strategy

Based on the preliminary analysis conducted in the previous section, we employ the panel cross-sectionally augmented ARDL model (CS-ARDL) proposed by Chudik et al. (2016). The CS-ARDL's flexibility accommodates three crucial features of the dataset: country-heterogeneity, cross-sectional dependence, and mixed integration order. Compared to the standard ARDL models, the CS-ARDL approach – which can be seen as an ARDL version of the dynamic common correlated estimator (DCCE) first introduced by Pesaran (2006) and then extended by Chudik and Pesaran (2015) – accounts for cross-sectional dependence by augmenting the model with the cross-sectional means of the dependent and independent variables (Chudik et al. 2016). We specify our panel CS-ARDL model as follows:

2 $${y}_{i,t}=\sum_{l=1}^{{k}_{y}}{\varphi }_{i,l}{y}_{i,t-l}+\sum_{l=0}^{{k}_{{\varvec{x}}}}{{\varvec{\beta}}\boldsymbol{^{\prime}}}_{i,l}{{\varvec{x}}}_{i,t-l}+\sum_{l=0}^{{k}_{{\varvec{w}}}}{{\varvec{\lambda}}\boldsymbol{^{\prime}}}_{i,l}{{\varvec{w}}}_{i,t-l}+\sum_{l=0}^{{k}_{{\varvec{z}}}}{{\varvec{\delta}}\boldsymbol{^{\prime}}}_{i,l}{\overline{{\varvec{z}}} }_{t-l}+{\alpha }_{i}+{\omega }_{i}\tau +{\varepsilon }_{i,t}$$

where $y$, ${\varvec{x}},$ and ${\varvec{w}}$ are sets of fiscal and non-fiscal variables defined according to Eq. 1, the vector ${\overline{{\varvec{z}}} }_{t}={({\overline{y} }_{t}, {\overline{{\varvec{x}}} }_{t},{\overline{{\varvec{w}}} }_{t})}^{^{\prime}}={({N}^{-1}\sum_{i=1}^{N}{y}_{it},{N}^{-1}\sum_{i=1}^{N}{{\varvec{x}}}_{it}, {N}^{-1}\sum_{i=1}^{N}{w}_{it})}^{^{\prime}}$ proxies the common correlated factors, the set of parameters **δ** denotes the country-specific factor loadings, ${\alpha }_{i}$ is the country-specific intercept, $\tau$ represents the linear time trend and ${\varepsilon }_{i,t}$ is the idiosyncratic error term. The parameters $\varphi$ and $\omega$, as well as the sets of parameters ${\varvec{\beta}}$, ${\varvec{\lambda}}$ and ${\varvec{\delta}}$, are heterogenous coefficients randomly distributed around a common mean with unit-specific noise.

In a dynamic context, the implied inclusion of lagged dependent variables on the right-hand side of the equation raises the problem of endogeneity. To mitigate such a bias, a strictly positive value of ${k}_{{\varvec{z}}}$ is needed. Following Chudik and Pesaran (2015), we tackle the endogeneity by setting ${k}_{{\varvec{z}}}=\sqrt[3]{T}$. The values of the parameters ${k}_{y}$, ${k}_{{\varvec{x}}}$ and ${k}_{{\varvec{w}}}$ – that define the lag structure of the observable variables – are chosen according to the Akaike Information Criterion (AIC).

MG, PMG, and dynamic fixed-effect (DFE) estimators are the main estimation techniques employed in dynamic heterogeneous models. The MG estimator allows for heterogeneous slope coefficients by computing time series regressions at the individual level and then averaging the coefficients. The PMG estimator can be seen as a combination of pooled and heterogenous estimations (Blackburne and Frank 2007; Ditzen 2018) as it allows for heterogeneity in the short-run but imposes homogeneity in the long-run. In this study, we do not employ DFE since its estimates are likely biased when short-run coefficients are heterogeneous across units, as discussed in Pesaran and Smith (1995). The homogeneity of long-run coefficients can be verified through the Hausman (1978) test. Under the null hypothesis of no systematic differences across the coefficients, the PMG estimator is more efficient than the MG estimator (Arnolod et al. 2011). Juodis et al. (2021) demonstrate that the consistency of pooled CCE estimators holds even when the unobserved common factors are correlated with the independent variables and greater than the number of regressors plus one.

The theoretical arguments proposed by Elmendorf and Mankiw (1999) – which place the dynamics of government activity and capital formation within a long-run perspective – motivate us to identify the short- and long-run effects and to investigate the cointegrating properties between government expenditure and private investments. As Ditzen (2021) demonstrates, the CS-ARDL model can be transformed into an ECM version while maintaining its inferential properties. Following his lines, we re-parametrise Eq. 2 as follows:

3 $$\Delta {y}_{i,t}={\xi }_{i}\left[{y}_{i,t-l}-{{{\varvec{\theta}}}^{\boldsymbol{^{\prime}}}}_{i}{{\varvec{x}}}_{i,t-1}-{{{\varvec{\phi}}}^{\boldsymbol{^{\prime}}}}_{i}{{\varvec{w}}}_{i,t-1}\right]+\sum_{l=1}^{{k}_{y}-1}{\varphi }_{i,l}{\Delta }_{l}{y}_{i,t-1}+\sum_{l=0}^{{k}_{{\varvec{x}}}-1}{{{\varvec{\beta}}}^{\boldsymbol{^{\prime}}}}_{i,l}{{\Delta }_{l}{\varvec{x}}}_{i,t}+\sum_{l=0}^{{k}_{{\varvec{w}}}-1}{{{\varvec{\lambda}}}^{\boldsymbol{^{\prime}}}}_{i,l}{{\Delta }_{l}{\varvec{w}}}_{i,t-l}+\sum_{l=0}^{{k}_{{\varvec{z}}}}{{\varvec{\delta}}\boldsymbol{^{\prime}}}_{i,l}{\overline{{\varvec{z}}} }_{t-l}+{\omega }_{i}\tau +{u}_{i,t}$$

where the long-run coefficients are computed as follows:

4 $${\widehat{{\varvec{\theta}}}}_{i}=\frac{\sum_{l=0}^{{k}_{{\varvec{x}}}}{\widehat{{\varvec{\beta}}}}_{i,l} }{1-\sum_{l=1}^{{k}_{{\varvec{y}}}}{\widehat{\varphi }}_{i,l}}, {\widehat{{\varvec{\phi}}}}_{i}=\frac{\sum_{l=0}^{{k}_{{\varvec{w}}}}{\widehat{{\varvec{\lambda}}}}_{i,l} }{1-\sum_{l=1}^{{k}_{{\varvec{y}}}}{\widehat{\varphi }}_{i,l}}$$

The parameter φ and the sets of parameters **β** and **λ** capture the short-run effects, Δ is the first difference operator, the square brackets contain the error correction term and ${\xi }_{i}$ denotes the speed of adjustment to the long-run equilibrium. In other words, the parameter ${\xi }_{i}$ measures the speed at which the economies converge to their long-run equilibrium paths following a shock. Therefore, the estimated coefficient associated with the error correction term is expected to be negative. The possibility of estimating a cointegrating relationship when the variables in the model are $I($ 0) or $I$(1) is discussed in the econometric literature (see, for instance, Pesaran et al. 2001; Lütkepohl 2005).

Implementing a formal cointegration test within the empirical design outlined in this study raises some concerns, given that *i*) the dependence across the units is strong and *ii*) the order of integration is mixed. Most of the existing cointegration tests require cross-sectional independence and nonstationarity in levels. The bound testing approach proposed by Pesaran et al. (2001) allows for a mixed order of integration – provided that the variables become stationary after being differentiated no more than once – but it applies to *univariate* time series analysis. Westerlund (2007) proposes a structural-based cointegration testing procedure for panel datasets allowing for heterogeneity, autocorrelation, and cross-sectional dependence.^6^ However, the Westerlund procedure assumes all the variables to be *I*(1). Another approach could be implementing the bounding test approach for each unit separately, but the power of the test would differ across the units as the panel employed in this paper is unbalanced. Despite that, we can rely on the statistical significance of the error correction term as sufficient evidence of a cointegrating relationship (see, for instance, Pesaran et al. 1999; Persyn and Westerlund 2008; Eberhardt and Presbitero 2015).

## Baseline estimates

In this section, we report and discuss the baseline estimates of Eq. 3, where the dependent variable is the logarithm of real private investments per capita. For all the specifications proposed in this article, we prefer the PMG estimates as we cannot reject the hypothesis of long-run homogeneity of the coefficients. The country-specific short-run coefficients and factor loadings of the baseline models are reported in the Appendix.

The results in Table 4 are relative to the GDP share of TGE and its bipartition into *productive* and *unproductive* categories. We can observe that TGE is negatively and significantly associated with private investments both in the short- and long-run, with a stronger impact in the latter case. When considering the GDP share of unproductive expenditure, the detrimental effect is higher. The evidence on productive government expenditure appears less clear than its counterpart, as both the sign and significance of the related coefficient change when output and interest rate enter the model. The error correction term is statistically significant across all the specifications, with the speed of adjustment to long-run equilibrium ranging from -0.3039 to -0.2216. We interpret such results as evidence of a cointegrating relationship between private investments and government expenditure. The *p*-values associated with Pesaran (2021) 's CD test are above 0.05, suggesting that the augmentation with current and lagged cross-sectional averages adequately account for cross-sectional dependence.

**Table 4:** CS-ARDL regressions

	(1)	(2)	(3)	(4)
Short-run coefficients				
TGE / GDP	–1.4075**		-0.6928*	
	(0.7067)		(0.3875)	
PGE / GDP		–0.3972		0.2688
		(0.7895)		(0.9817)
UGE / GDP		–3.7026***		–2.1454*
		(1.0385)		(1.2377)
GDP			0.2371	0.4230
			(0.5374)	(0.3693)
INTEREST			–0.0051**	–0.0029*
			(0.0025)	(0.0019)
Long-run coefficients				
TGE / GDP	–6.3522***		–4.2421***	
	(0.7067)		(0.3875)	
PGE / GDP		–1.3070*		0.9101
		(0.7895)		(0.9817)
UGE / GDP		–12.1839***		–7.2639***
		(1.0385)		(1.2377)
GDP			1.4519***	1.4322***
			(0.5374)	(0.3693)
INTEREST			–0.0028	–0.0053
			(0.0055)	(0.0055)
ECT(*t-1*)	–0.2216**	–0.3039**	–0.2633*	–0.2954**
	(0.1051)	(0.1462)	(0.1483)	(0.1310)
N*T	608	608	489	489
Groups	28	28	25	25
CD *p*-value	0.696	0.0815	0.380	0.105
Hausman *p*-value	0.869	0.089	0.743	0.116

Notes: PMG estimates. Dependent variable: Real private investments per capita (logarithm). Linear country-specific time trends are included in the models but not reported. The standard errors (reported in square brackets) are calculated through the delta method. The *p*-values of both cross-sectional dependence and Hausman tests are reported. The asterisks ***, **, * denote significance at 1%, 5%, 10%, respectively. The optimal lag length is selected according to the value that minimises AIC

The evidence of the detrimental effect of total government expenditure on the long-run dynamics of private investments per capita is broadly consistent with Aschauer (1989), Barro (1991), Alesina et al. (2002), Blanchard and Perotti (2002) and Furceri and Sousa (2011). Such a negative long-run relationship is stronger when we consider the GDP share of unproductive expenditure, consistently with Argimon et al. (1997). Unlike the main outcomes of Afonso and Aubyn (2019), increases in productive spending seem not to have either a crowding-in or crowding-out effect on private investments.

The results in Table 5 provide insights into the response of private investments to the reallocation of public resources towards productive or unproductive government expenditure, as the size of government intervention relative to GDP does not adjust in the model. Since we now consider the TGE share of productive and unproductive categories, we estimate their effects in separate regressions as they are perfectly multicollinear. Although the previously discussed estimates (reported in Table 4) suggest that increases in productive government expenditure are not significantly associated with variations in private investments, we can interestingly notice that the reallocation of public resources towards productive public expenditure has a significant and positive impact on private investments, with higher coefficients related to the long-run. Conversely, the estimates show a significant opposite effect when the share of unproductive government expenditure increases. This result is intuitive as a one-unit increase in the *productive* category reflects the opportunity cost in terms of *unproductive* expenditure and vice-versa, provided that the total government expenditure is constant. Our results align with Gemmell et al. (2016) and Chu et al. (2020), keeping in mind that they consider GDP as dependent variable rather than private investment. It is worth observing that the real interest rate is robustly associated with both short- and long-run adverse effects on private investments. Such a finding could be pivotal in the economic interpretation of the results, as the real interest rate typically represents one of the main transmission channels linking government activity to the private sector (see, for instance, Atesoglu and Emerson 2008). The speed of adjustment to deviation from the long-run equilibrium does not differ considerably compared to the estimates in Table 4.

**Table 5:** CS-ARDL regressions

	(1)	(2)	(3)	(4)
Short-run coefficients				
TGE / GDP	–1.7157***	–1.7012***	–0.6801*	–0.7291**
	(0.5962)	(0.6231)	(0.3713)	(0.3698)
PGE / TGE	1.6403***		1.2185	
	(0.6164)		(1.0571)	
UGE / TGE		–1.5142**		–1.2502
		(0.5854)		(1.0289)
GDP			0.4826	0.4730
			(0.3194)	(0.3550)
INTEREST			-0.0049**	-0.0033*
			(0.0023)	(0.0021)
Long-run coefficients				
TGE / GDP	–5.5561***	–5.6710***	–2.1134***	-2.3279***
	(0.5962)	(0.6231)	(0.3713)	(0.3698)
PGE / TGE	5.3122***		3.7863***	
	(0.6164)		(1.0571)	
UGE / TGE		–5.0477***		–3.9920***
		(0.5854)		(1.0289)
GDP			1.4996***	1.5103***
			(0.3194)	(0.3550)
INTEREST			-0.0031*	–0.0046*
			(0.0018)	(0.0025)
ECT(*t-1*)	–0.3088**	–0.3000*	–0.3218**	–0.3132*
	(0.1399)	(0.1529)	(0.1454)	(0.1572)
N*T	608	608	489	489
Groups	28	28	25	25
RMSE	0.0566	0.0570	0.0513	0.0517
CD *p*-value	0.0713	0.0513	0.108	0.109
Hausman *p*-value	0.632	0.115	0.454	0.090

Notes: PMG estimates. Dependent variable: Real private investments per capita (logarithm). Linear country-specific time trends are included in the models but not reported. The standard errors (reported in square brackets) are calculated through the delta method. The *p*-values of both cross-sectional dependence and Hausman tests are reported. The asterisks ***, **, * denote significance at 1%, 5%, 10%, respectively. The optimal lag length is selected according to the value that minimises AIC

## Government budget constraint

This section investigates whether the financing method of government expenditure matters for the dynamics of private investments. Such analysis requires a broader examination of fiscal policy. For this purpose, the most straightforward way is to consider the government budget constraint (GBC) introduced by Miller and Russek (1997), not rarely employed in the literature (e.g., Kneller et al. 1999; Ahmed and Miller 2000; Ahmed and Miller 2000; Gemmell et al. 2011; Gemmell et al. 2011; Chu et al. 2020). However, Gemmell et al. (2016) argue that most related articles do not consider GBC even when the latter could boost the consistency of the analyses. The use of GBC appears to be even more scarce when analysing the private investments' effects of public spending.^7^

In order to construct a GBC system that fits our empirical framework and data, we start with the following condition:

5 $${TGE}_{i,t}\equiv {REVENUES}_{i,t}+{DEFICIT}_{i,t}$$

The identity specified in Eq. 5 states that government revenues and public debt finance the current government expenditure. The financing method likely affects the dynamics of investments as the distortive effects of tax and debt involve different time horizons and, consequently, how agents' expectations are affected. For instance, Ahmed and Miller (2000) classify the financing sources into taxes and public debt, whereas Gemmell et al. (2011) and Gemmell et al. (2011) consider distortionary taxes, non-distortionary taxes and public debt. In this study, we consider public debt, tax revenue, and non-tax revenue.^8^ Unlike tax-revenue and public debt, non-tax revenue normally does not imply a current or future fiscal burden on taxpayers. Therefore, it is worth analysing whether financing public spending through non-tax revenue is significantly less distortive for the private sector.^9^

The GBC system specified in Eq. 5 can be extended as follows:

6 $${TGE}_{i,t}\equiv {TAX REVENUE}_{i,t}+NON{TAX REVENUE}_{i,t}+{DEFICIT}_{i,t}$$

By dividing both sides of the equation by GDP, we obtain the GBC system relative to the aggregate output. Equation 6 describes a mathematical identity as a unit increase on the left-hand side of the equation is balanced by a unit decrease on the right-hand side and vice-versa. The identity properties of Eq. 6 are pivotal in identifying whether a unit increase in government expenditure is financed via tax revenue, non-tax revenue, public debt or a combination of these three alternatives. The financing variables that do not enter the regression represent the method employed to finance public spending, as they adjust in the model following a unit variation in government expenditure. The estimates that include the GBC system only consider the GDP shares of the expenditure variables since the TGE share of productive and unproductive categories are not affected by changes in the composition of the financing variables by construction.

The short-run effect of TGE is negative and statistically significant only when financed by tax revenue or a combination of tax and non-tax revenue (Table 6). In the long-run, TGE adversely and significantly affects the dynamics of private investments per capita regardless of how it is financed, therefore not differing from the previous estimates in which financing restrictions are not imposed. However, we should point out that the absolute value of the long-run estimated parameters is higher when spending is financed via deficit, consistent with Gemmell et al. (2011). Financing government expenditure via non-tax revenue seems to be the less detrimental alternative for the long-run dynamics of private investment. Such a finding is likely due to the non-distortionary nature of non-tax revenue. Compared to the regressions in which no GBC is imposed, the speed of adjustment to the long-run equilibrium is lower, ranging from -0.1942 to -0.1506.

**Table 6:** CS-ARDL regressions

Financing category:	Deficit	Tax and non-tax revenue	Deficit and tax revenue	Tax revenue	Non-tax revenue
	(1)	(2)	(3)	(4)	(5)
Short-run coefficients					
TGE / GDP	–0.5817	–1.3400**	–0.8231	–1.1611**	–1.5666
	(0.6448)	(0.5251)	(0.5897)	(0.5622)	(0.9632)
GDP	0.3187	0.3132	0.2390	0.3187	0.3187
	(0.5087)	(3.3024)	(0.4414)	(0.5087)	(0.5087)
INTEREST	–0.0006	–0.0007	–0.0004	–0.0006	–0.0006
	(0.0066)	(0.0569)	(0.0070)	(0.0066)	(0.0066)
TAX REVENUE	–0.5794				0.4055
	(0.7982)				(1.0377)
NON-TAX REVENUE	–0.9849		–0.7703	–0.4055	
	(0.7814)		(0.9544)	(1.0303)	
DEFICIT		0.7340		0.5794	0.9849
		(0.5995)		(0.7950)	(0.7814)
Long-run coefficients					
TGE / GDP	–8.5485***	–5.2389***	–7.3906***	–6.3356***	–3.1741***
	(0.9632)	(0.5897)	(0.5251)	(0.5622)	(0.6448)
GDP	1.2051**	1.2298	1.3886***	1.2051**	1.2051**
	(0.5087)	(3.3024)	(0.4414)	(0.5087)	(0.5087)
INTEREST	–0.0021*	–0.0026**	–0.0023*	–0.0021*	–0.0015*
	(0.0013)	(0.0012)	(0.0013)	(0.0014)	(0.0009)
TAX REVENUE	–3.1614***				2.2129**
	(0.7982)				(1.0377)
NON-TAX REVENUE	–5.3744***		–4.9028***	–2.2129**	
	(0.7814)		(0.9544)	(1.0303)	
DEFICIT		4.0482***		3.1614***	5.3744***
		(0.5995)		(0.7950)	(0.7814)
ECT(*t-1*)	–0.1833*	–0.1813**	–0.1571**	–0.1942**	–0.1506**
	(0.1068)	(0.0844)	(0.0775)	(0.0959)	(0.0702)
N*T	555	555	555	555	555
Groups	28	28	28	28	28
RMSE	0.0618	0.0619	0.0623	0.0618	0.0618
CD p-value	0.0676	0.0556	0.130	0.0503	0.0612
Hausman *p*-value	0.353	0.156	0.088	0.054	0.863

Notes: PMG estimates. Dependent variable: Real private investments per capita (logarithm). Linear country-specific time trends are included in the models but not reported. The standard errors (reported in square brackets) are calculated through the delta method. The *p*-values of both cross-sectional dependence and Hausman tests are reported. The asterisks ***, **, * denote significance at 1%, 5%, 10%, respectively. The optimal lag length is selected according to the value that minimises AIC

Table 7 reports an analogue exercise in which the TGE is decomposed into productive and unproductive components. Consistently with the outcomes reported in the previous section, no robust evidence for a significant effect of productive expenditure arises, although the long-run coefficients are statistically significant at the 10% level when it is totally or partially financed via debt. Conversely, the long-run effect of unproductive expenditure is negative and significant across all the specifications, suggesting that the way it is financed does not affect the sign or statistical significance of the associated coefficients. In addition, the long-run effect of unproductive expenditure appears to be slightly worse when a deficit enlargement accompanies it. However, the short-run effects of unproductive spending significantly threaten private investments only when a rise in tax revenue or a combination of non-tax and tax revenue is involved. Such a finding appears particularly interesting as it suggests that, under certain circumstances, the supply side of the private sector can tolerate government intervention aimed at non-growth purposes – at least in the short-run. The bipartition into productive and unproductive expenditure implies a faster adjustment to the long-run equilibrium, as the coefficients associated with the error correction term range from -0.5971 to -0.5567.

**Table 7:** CS-ARDL regressions

Financing category:	Deficit	Tax and non-tax revenue	Deficit and tax revenue	Tax revenue	Non-tax revenue
	(1)	(2)	(3)	(4)	(5)
Short-run coefficients					
PGE / GDP	–0.0292	–0.2633	–0.6151	–0.5729	0.1917
	(1.5802)	(0.7765)	(2.5358)	(1.6147)	(3.6432)
UGE / GDP	–2.2379	–2.8538*	–3.3884	–2.7796*	-2.4015
	(3.8165)	(1.5057)	(5.4492)	(1.7082)	(4.8032)
GDP	0.7930	1.1554	0.8091	0.7832	0.7332
	(1.0117)	(9.5032)	(6.5905)	(1.1861)	(1.2535)
INTEREST	–0.0092	–0.0091	–0.0089	–0.0088	–0.0093
	(0.0236)	(0.1177)	(0.0951)	(0.0328)	(0.0327)
TAX REVENUE	–0.5224				–0.3594
	(1.4106)				(3.9283)
NON-TAX REVENUE	–0.1501		0.6755	0.3873	
	(2.9201)		(3.4272)	(3.4604)	
DEFICIT		–0.5074		0.5440	0.1710
		(0.6804)		(1.1781)	(2.7709)
Long-run coefficients					
PGE / GDP	–0.0525*	–0.4558	–1.0300*	–1.0281	0.3442
	(0.0332)	(0.7765)	(0.6358)	(1.6147)	(3.6432)
UGE / GDP	–6.0148**	–5.0156*	–5.4059*	–5.3197*	–4.9656*
	(3.0211)	(3.4080)	(3.4352)	(3.2009)	(3.0688)
GDP	0.8335	1.5419	1.0330	0.8283	0.7638
	(1.0117)	(9.5032)	(6.5905)	(1.1861)	(1.2535)
INTEREST	–0.0096*	–0.0121*	–0.0113*	–0.0094*	–0.0096*
	(0.0060)	(0.0077)	(0.0061)	(0.0058)	(0.0053)
TAX REVENUE	–0.9384				–0.6452
	(1.4106)				(3.9283)
NON-TAX REVENUE	–0.2697		1.1312	0.6951	
	(2.9201)		(3.4272)	(3.4604)	
DEFICIT		–0.8784		0.9764	0.3070
		(0.6804)		(1.1781)	(2.7709)
ECT(*t-1*)	–0.5567*	–0.5776***	–0.5971**	–0.5572*	–0.5570**
	(0.3258)	(0.1612)	(0.2663)	(0.3420)	(0.2565)
N*T	428	445	445	428	428
Groups	21	22	22	21	21
RMSE	0.0402	0.0443	0.0442	0.0402	0.0402
CD p-value	0.0086	0.0550	0.0503	0.0088	0.0087
Hausman *p*-value	0.108	0.471	0.166	0.238	0.599

Notes: PMG estimates. Dependent variable: Real private investments per capita (logarithm). Linear country-specific trends are included in the models but not reported. The standard errors (reported in square brackets) are calculated through the delta method. The *p*-values of both cross-sectional dependence and Hausman tests are reported. The asterisks ***, **, * denote significance at 1%, 5%, 10%, respectively. The optimal lag length is selected according to the value that minimises AIC

With due parsimoniousness, we can argue that short-run dynamics of private investments are negatively affected by tax-financed expenditure, while long-run dynamics seem to be mainly threatened by deficit-financed spending – consistent with Elmendorf and Mankiw (1999). However, the results discussed in this section suggest that the crowding-out effect is robust only when total and unproductive expenditures are considered, whereas no robust evidence of crowding-in or crowding-out effect of productive government expenditure emerges.

## Robustness check

This section carries out additional empirical exercises. We parsimoniously implement changes involving both the explanatory variables and inferential spheres, consistently with this study's economic and econometric frameworks.

Firstly, we assess the behaviour of the coefficients of main interest by modifying the compositions of total, productive and unproductive expenditure. For that purpose, we consider some of the most influential single components for private investments and exclude them from the spending categories defined in this study. The exclusion of the influential variables is based on the results at disaggregated levels that emerged in Carvelli (2022). Therefore, we exclude spending on social protection as it concerns the unproductive category (Table 8, columns 1–4) and defence and health regarding the productive category (Table 8, columns 5–8).

**Table 8:** CS-ARDL regressions

	Exclusion of social protection				Exclusion of social protection, defence and health			
	AIC		AIC + 1		AIC		AIC + 1	
	(1)	(2)	(3)	(4)	(5)	(6)	(7)	(8)
MODEL A								
Short-run coefficients								
PGE / GDP	–0.5393		–0.4808		–0.5685		–0.4401	
	(0.5378)		(1.1639)		(0.4983)		(0.8267)	
UGE / GDP	–1.3131*		–1.4082		–1.7842*		–1.0055	
	(0.6709)		(8.7978)		(0.9094)		(7.5477)	
Long-run coefficients								
PGE / GDP	–1.6089***		–4.8482***		–1.9337***		–5.3797***	
	(0.5378)		(1.7280)		(0.4983)		(1.5589)	
UGE / GDP	–3.2652***		–5.9731***		–3.6052***		–6.1805***	
	(0.8505)		(1.3467)		(0.7619)		(1.1120)	
MODEL B								
Short-run coefficients								
TGE / GDP	–2.7743***	–2.8114***	–2.4877	–2.5373	–3.1714***	–3.2610***	–2.5478***	–2.6379***
	(0.7439)	(0.7285)	(1.8036)	(2.0434)	(0.8414)	(0.8509)	(0.8739)	(0.8962)
PGE / TGE	3.1233***		2.9054***		3.5067***		2.7450***	
	(1.1646)		(0.7693)		(1.1846)		(0.9774)	
UGE / TGE		–3.1233***		–2.8353***		–3.5699***		–2.7390***
		(1.0741)		(0.8738)		(1.1094)		(0.9295)
Long-run coefficients								
TGE / GDP	–7.7683***	–7.8060***	–13.0381***	–14.9323***	–8.4267***	–8.5715***	–11.6052***	–12.4032***
	(0.7439)	(0.7285)	(2.7457)	(2.8123)	(0.8414)	(0.8509)	(1.6419)	(1.6741)
PGE / TGE	6.3827***		11.5152***		6.8788***		9.3617***	
	(1.3047)		(3.8369)		(1.2377)		(2.0414)	
UGE / TGE		–5.7472***		–11.6945***		–6.5714***		–9.3501***
		(1.1519)		(3.4083)		(1.0881)		(1.9501)

Notes: PMG estimates. Dependent variable: Real private investments per capita (logarithm). GDP, INTEREST, and linear country-specific time trends are included in the models but not reported. The standard errors (reported in square brackets) are calculated through the delta method. The *p*-values of both cross-sectional dependence and Hausman tests are reported. The asterisks ***, **, * denote significance at 1%, 5%, 10%, respectively. The optimal lag length is selected according to the value that minimises AIC

Secondly, we assess the estimates' sensitivity to the lag structure of the variables by estimating a set of regressions whose optimal lag order, selected according to the value that minimises AIC, is increased by one period.

Thirdly, we modify the set of control variables. The real interest rate is replaced with the real exchange rate to control for the economic and financial interactions at the international level. Moreover, following Ahmed and Miller (2000), we add as a covariate the international openness index – defined as the sum of export and imports over GDP – since the links between private investments and real exchange likely depend on the degree of openness of the economies (Servén 2003). The robust role of international trade in explaining investment variations is widely discussed in Levine and Renelt (1992). We made sure that the integration order of the new control variables is lower than two (according to the CIPS test, real exchange rate and openness are *I*(0) and *I*(1), respectively). The results are not reported but are available upon request.

The estimated parameters of the key fiscal variables closely align with their counterparts in the baseline estimates, regardless of whether the restrictions on the financing sources are imposed. Interestingly, the unproductive government expenditure still crowds-out private investments significantly, although it is now net of the social protection component. Similarly, excluding spending on defence and health does not yield different long-run coefficients in terms of sign and statistical significance. Such findings suggest that the individually insignificant components might become significant if jointly considered or grouped as a unique category, highlighting that the effects of fiscal policy may be shaped by complex interrelations among the fiscal variables.

## Conclusions

In this paper, we have re-examined the relationship between government expenditure and private investments by exploiting renewed fiscal time series for 28 OECD countries over 1990–2019. We build the analysis on a dynamic heterogeneous framework that accommodates some relevant characteristics of the panel, such as nonstationarity, country-heterogeneity, and cross-sectional dependence. Such econometric issues and the way public spending is financed have been scarcely addressed in the related literature. The main contributions of this study can be summarised as follows: a) we account for cross-sectional dependence and establish long-run cointegrating relationships by estimating an ECM version of the novel CS-ARDL model; b) we construct a government budget constraint system (GBC) to condition the effects of government expenditure to the financing variables; c) we make a bipartition of government expenditure into *productive* and *unproductive*, considering them as shares of both GDP and total government expenditure. Several remarks are in order.

Firstly, the crowding-out effect of government expenditure on private investments is significant and robust across all the fiscal financing methods and becomes more intense in the long-run. Secondly, level increases in productive government expenditure are neutral for the dynamics of private investments, but a reallocation of public resources towards productive spending is robustly associated with positive long-run effects. Thirdly, both level increases and reallocations of public resources towards unproductive government expenditure are robustly associated with a fall in private investments. Fourthly, the estimates suggest that the financing methods may affect the significance of the estimated coefficients, and their influence varies according to the spending categories. The results that arose in this article highlight that the most frequent case is that short-run crowding-out effects are tendentially associated with tax-financed expenditure, whereas long-run crowding-out effects mainly arise with deficit-financed expenditure.

The findings that emerged in this paper might have important implications for fiscal policy in advanced economies.

The effects of government activity on capital formation in the private sector vary according to the way public resources are allocated, the financing method employed and the time horizon considered. Neglecting such features in the analyses might lead to misleading conclusions and inefficient policy measures, even when fiscal actions are not motivated by growth objectives.

## Data Availability

The dataset generated and analysed in the current article is available upon reasonable request.

## Appendix

Table

**Table 9:** Short-run country-specific coefficients

Country	TGE/GDP	PGE/GDP	UGE/GDP	PGE/TGE	UGE/TGE
Austria	1.978	2.490	–3.829	3.052	0.926
Belgium	–0.331	–0.759	–3.645	1.428	–1.649
Czech Republic	–0.642	0.457	–1.749	0.695	–0.227
Denmark	2.091	1.810	0.656	0.199	–0.432
Estonia	0.128	0.583	20.037	–6.087	5.170
Finland	–1.063	–3.328	–0.596	2.075	–0.101
France	–2.512	–1.133	–1.863	0.721	–0.589
Germany	0.693	3.821	–4.208	1.142	–0.843
Greece	0.595	1.072	6.504	4.766	–2.936
Hungary	1.374	1.713	2.238	2.245	–1.026
Iceland	–2.666	–6.037	–10.216	6.257	–6.239
Ireland	3.240	–2.074	6.378	–5.983	5.710
Italy	–1.940	–0.456	–1.746	2.035	–2.286
Japan	–1.554				
Latvia	1.540	–6.609	14.889	–6.322	5.227
Lithuania	0.736	–1.313	–0.783	–0.131	0.537
Luxembourg	–0.449	6.949	0.170	4.608	–2.289
Netherlands	–1.543	–11.296	–11.272	–10.486	3.746
Norway	0.385	–0.685	7.099	–3.100	2.679
Poland	–1.461	–0.521	–0.591	–0.113	–0.543
Portugal	0.848	–0.746	0.176	–0.765	1.977
Slovakia	–1.469	–1.160	–4.494	–0.121	–0.940
Slovenia	0.960	0.570	2.556	1.972	–0.782
Spain	0.410	1.401	4.709	–2.769	1.943
Sweden	–0.246	2.554	2.871	–0.832	1.083
Switzerland	–0.345	1.576	0.080	0.397	–0.241
United Kingdom	–0.641	2.706	–3.969	3.615	–4.029
United States	–3.398	0.440	–6.469	0.223	–6.289

Notes: Short-run country-specific coefficients, PMG estimates. The estimated coefficients refer to the baseline regressions without non-fiscal controls

9

Table

**Table 10:** Country-specific factor loadings

Country	Cross–sectional averages proxying common correlated factors					
	INV	PGE/GDP	UGE/GDP	PGE/TGE	UGE/TGE	PGE/GDP
Austria	–0.003	–2.541	–2.424	–0.748	–1.319	2.306
Belgium	0.466	–0.357	–0.730	0.563	–2.454	2.974
Czech Republic	0.535	0.503	4.875	2.184	–0.736	–0.271
Denmark	0.643	2.099	–3.422	8.875	–3.307	3.832
Estonia	1.957	5.820	–11.134	34.254	–16.127	16.819
Finland	–0.097	–1.848	–2.330	1.815	–2.218	2.761
France	0.012	–1.256	–1.406	–0.015	–2.051	1.128
Germany	0.453	–0.824	–3.529	0.734	–2.370	2.884
Greece	0.933	–0.117	–9.785	1.649	2.061	2.971
Hungary	1.152	3.628	–2.173	6.247	4.749	–3.097
Iceland	3.775	–5.489	–8.522	–37.241	–2.428	–3.626
Ireland	1.534	6.200	–26.052	62.467	–15.306	3.645
Italy	–0.106	–0.899	0.325	1.577	–0.138	–0.020
Japan	0.514	–1.017				
Latvia	0.501	–6.219	–3.893	–32.405	1.695	1.096
Lithuania	3.314	10.550	14.120	0.536	4.634	–2.566
Luxembourg	0.136	1.341	–0.948	–23.282	7.383	–6.271
Netherlands	–0.138	2.446	–9.410	24.981	–6.232	5.771
Norway	1.431	6.245	–3.803	7.806	–6.547	5.853
Poland	0.699	0.581	6.457	3.345	–2.230	3.338
Portugal	0.517	–0.128	–3.440	5.615	–2.157	4.474
Slovakia	2.052	8.783	22.777	7.301	8.705	–6.192
Slovenia	0.562	–4.190	–3.236	–8.445	3.693	–1.725
Spain	0.429	–2.427	0.344	–9.450	2.396	–1.623
Sweden	0.066	–2.120	5.170	–2.798	2.632	–1.650
Switzerland	0.493	0.788	–1.361	1.789	–0.600	0.337
United Kingdom	0.708	–0.847	–1.311	1.442	0.532	0.442
United States	0.174	0.391	1.518	–3.553	–0.799	–3.851

Notes: Country-specific factor loadings, PMG estimates. The estimated coefficients refer to the baseline regressions without non-fiscal controls

10

## Footnotes

1 See IMF *Fiscal Monitor* (April, 2022).

2 In fact they find a very small fiscal multiplier. This might be due to the opposite sign effect of government expenditure on aggregate output and private investments.

3 For instance, government expenditure represents around 42% of GDP in OECD area in 2019.

4 See for, among others, Pesaran (2006), Eberhardt (2012), Chudik and Pesaran (2015), Chudik et al. (2016).

5 The use of *primary* deficit/surplus is advantageous as it is net of interest payments.

6 The author employs a bootstrap approach to accommodate cross-sectional dependence.

7 To the best of our knowledge, GBC system is adopted only in Ahmed and Miller (2000) when employing private investments as dependent variable.

8 In order to maintain sufficiently long time series for a large number of countries, we do not disaggregate tax data in its components due to the limited length of some tax-specific time series.

9 For instance, Gemmell et al. (2011) find that distortionary tax-financed spending has the worst growth effects, followed by deficit (intermediate growth effects) and non-distortionary taxes (best growth effect).
